# Does Time and Experience Matter in Pediatric Arterial Ischemic Stroke (AIS) Intervention in Patients with an Initial Clinical Presentation of Mild/Moderate Severity? Long-Term Follow-Up Experience of a Single Tertiary Clinic

**DOI:** 10.3390/children12040407

**Published:** 2025-03-24

**Authors:** Gulten Ozturk, Erhan Biyikli, Olcay Unver, Omer Dogru, Evrim Karadag Saygi, Feyyaz Baltacioglu, Dilsad Turkdogan

**Affiliations:** 1 Pediatric Neurology Department, Marmara University Medical Faculty Pendik Research and Training Hospital, Istanbul 34899, Turkey; olcaymd@hotmail.com (O.U.); dturkdogan@hotmail.com (D.T.); 2 Radiology Department, Marmara University Medical Faculty Pendik Research and Training Hospital, Istanbul 34899, Turkey; biyiklierhan@hotmail.com; 3 Pediatric Hematology Department, Marmara University Medical Faculty Pendik Research and Training Hospital, Istanbul 34899, Turkey; drdogru@hotmail.com; 4 Physical Medicine and Rehabilitation Department, Marmara University Medical Faculty Pendik Research and Training Hospital, Istanbul 34899, Turkey; evrimkaradag4@hotmail.com; 5 Interventional Radiology Department, Marmara University Medical Faculty Pendik Research and Training Hospital, Istanbul 34899, Turkey; feyyazb@amerikanhastanesi.org

**Keywords:** ischemic stroke, pediatric, long term, outcome

## Abstract

Introduction: This study presents long-term data of pediatric AIS patients with a favorable initial clinical presentation who were followed by a tertiary pediatric neurology clinic with a well-organized stroke team. Method: Patients who were diagnosed with AIS at pediatric age (28 days–18 years) and followed for at least 5 years by the same clinic were included in this study. The clinical and demographical characteristics of the patients were retrospectively collected from their medical records. At their last visit, the modified Rankin scale (mRS) and Pediatric Stroke Outcome Measure Short Neuro Exam (PSOM-SNE) were administered, and a neurological examination was performed. Results: A total of 32 patients (20 of whom were male, 62.5%) were included in this study. Their mean age at the time of the study was 162.62 ± 64.4 (62–300) months. The mean age at first ischemic stroke was 77.39 ± 61.93 (0.5–180) months, and the mean follow-up duration was 85.44 ± 20.52 (60–121) months. Seventeen patients (53.3%) reported normal daily functions at the last visit. A younger presentation age (≤60 months) was related to a longer hospital admission duration (24 h vs. 9 h) and worse long-term functional outcomes (*p* = 0.023). The affected vascular territory did not have any significant effect on long-term clinical outcomes (*p* = 0.550). Anticoagulant treatment alone was consistent with a worse prognosis compared to antithrombotic treatment alone or the combination of both (*p* = 0.026). PSOM-SNE scores were helpful in detecting some mild cognitive and language dysfunctions in patients with favorable mRS scores and subtle neurological sequelae. Conclusions: Pediatric AIS with a mild presentation has some degree of long-term morbidity, even when handled at well-organized stroke centers. A younger presentation age has the highest risk of long-term neurological sequelae.

## 1. Introduction

Acute stroke is defined as a focal insult to one or multiple parts of the brain, which results in an acute neurological deficit lasting for more than 24 h and/or with radiological evidence [1]. An acute neurological deficit that lasts for less than 24 h is considered a transient ischemic attack (TIA) in the absence of radiological evidence [2].

Pediatric stroke is classified as either perinatal (0–28 days) or childhood (28 days–18 years) stroke, and 50% of cases are estimated to be ischemic in origin [3,4,5].

The incidence of acute pediatric ischemic stroke is estimated to be 2.6–3.3/100,000, and, although rare, it is an important cause of childhood mortality [6,7]. The broad spectrum of nonspecific symptoms at presentation mostly delays early suspicion and intervention.

Arterial ischemic stroke (AIS) in the pediatric population is more commonly seen under 5 years of age, and its etiology differs from that in the adult population [7]. It is known that pediatric stroke has a variable prognosis according to the etiology and age of incidence [8].

Although an ancient widespread belief was that childhood stroke has an overall better prognosis than adult stroke, with brain plasticity being the main factor responsible for this outcome, recent study results have provided data conflicting with this hypothesis. Recent studies have reported motor deficits in 50–62% of pediatric AIS cases, with only 30% of cases achieving long-term normal outcomes [8]. A younger age during the insult has been found to be one of the major risk factors for a poor long-term prognosis, and, considering the lifelong treatment and care burden on society, researchers have focused on the development of new follow-up and treatment strategies to improve the prognosis and decrease the sequelae [9].

As clinical studies have revealed the difference between pediatric stroke and adult stroke in terms of etiology and prognosis, consensus guidelines for the acute management of pediatric stroke have been regularly updated with the addition of new knowledge [10,11,12,13].

Pediatric stroke-related morbidity and mortality studies have revealed that both the short- and long-term outcomes have improved in recent years with advances in diagnoses and treatment options [14,15].

It has been proven that the presence of a well-organized, equipped, and experienced pediatric stroke team that applies the most up-to-date treatment algorithms in a pediatric emergency unit provides a great advantage in the short-term outcomes of pediatric stroke [16].

Our center is a tertiary clinic with a well-organized pediatric stroke intervention team, with the contribution of pediatric, pediatric neurology, pediatric neurosurgery, pediatric physical medicine and rehabilitation, interventional pediatric radiology, pediatric hematology (on call), and pediatric cardiology (on call) departments. This study aims to present the long-term outcomes of pediatric AIS patients with a mild presentation, whose initial intervention was performed by a well-organized stroke team and who were followed by a tertiary pediatric neurology clinic.

## 2. Methods

### 2.1. Study Cohort-Inclusion and Exclusion Criteria

The medical records of all pediatric patients diagnosed with acute stroke within an age range of 28 days to 18 years between the years of 2011 and 2018 at Marmara University Pediatric Neurology Clinic were retrospectively evaluated. Out of 60 patients, 32 with clinical and radiological evidence of AIS, whose first medical intervention was performed in the Marmara University Pediatric Emergency Unit and who had been followed for at least 5 years after initial diagnoses, were included in this study.

Patients who were referred to our clinic from different centers after their initial stabilization and/or whose physical rehabilitation follow-ups were performed at different centers were excluded from this study. Patients with severe clinical conditions at admission who stayed in the pediatric intensive care unit for more than 24 h or with a Glasgow coma scale score of less than 13 points were also excluded from this study. Patients with an underlying metabolic disease presenting with stroke were not included in this study.

### 2.2. Study Protocol

AIS was defined as the acute onset of neurological symptoms with evidence of acute infarction in an arterial territory in diffusion MRI, consistent with the patient’s clinical presentation. Hemorrhagic stroke was excluded using cranial computed tomography (CT) in the emergency room, and AIS was verified in all patients via further radiological interventions after the stabilization of their clinical status. Cranial MRI (which included axial T2, axial and coronal T2/flair, axial T1-weighted, and DWI sequences) was performed in all patients. All images were obtained using a 1.5 Tesla or 3 Tesla MRI device (Avanto and Verio, Siemens Healthcare, Erlangen, Germany). Cranial MRI angiography (time of flight), cervical MRI angiography with a gadolinium injection, and MRI venography were obtained if necessary. All radiological images were re-evaluated by the same neuroradiologist.

The neurological symptoms at admission, the time interval passing from symptom onset to hospital admission, the radiological findings at admission, and the investigations and tests performed during the hospital stay or in the follow-up were noted. Initial stroke age was subdivided into two groups, namely, early (≤60 months) and late (>61 months), considering the fact that the synaptogenesis and pruning process of the brain is mostly completed around 4 to 6 years of age, and AIS is more common under 5 years of age [7,17].

All patients had undergone baseline blood tests at hospital admission, including a complete blood count and blood glucose, electrolyte, and C-reactive protein levels. Echocardiography was performed in all patients within the first 24 h of their admission. All patients with no overt etiology for acute stroke underwent the tests included in the pediatric stroke investigation protocol of our center, including the following: ESR, protein C, protein S, factor V Leiden, antithrombin III, prothrombin gene mutation, lipoprotein A level, homocysteine, MTHFR gene mutation, factor 8 level, antiphospholipid IgM and IgGM, metabolic disease screening tests (TANDEM-MS, blood and urine amino acids, urine organic acids, blood and cerebrospinal fluid (CSF) lactate, and pyruvate), CSF varicella zoster PCR, cerebrospinal fluid (CSF) investigation for central nervous system (CNS) infection, blood C3, C4, RF, anti-dsDNA, cANCA, pANCA, ANA, anti-Ro, and anti-La.

Patients who regularly attended the pediatric neurology outpatient follow-up at least every 6 months, adhered to the prescribed medications, had any motor deficit at presentation, and attended their appointment dates at the physical medicine and rehabilitation clinic were considered to be followed regularly.

All of the patients included in this study had undergone a treatment protocol prepared by the pediatric stroke team of the center according to their underlying etiology, predisposing risk factors, and clinical status.

### 2.3. Outcome Measure Tests

All the patients included in this study were evaluated by the same pediatric neurologist investigator who performed the neurological examination, and they underwent a Pediatric Stroke Outcome Measure Short Neuro Exam (PSOM-SNE)-Child version (over 2 years of age) [17,18]. PSOM-SNE is an easily applicable objective outcome measure test that contains 114 items; it allows the physician to evaluate sensorimotor (right/left), language, and cognitive/behavior deficits [19]. Cognitive dysfunction was defined as the presence of poor school performance, language deficits, and attention deficits interfering with routine activities or the need to be followed by a child psychiatrist or psychologist.

The modified Rankin scale (mRS) was used for the classification of functional outcomes, with an mRS score of 0–1 forming the favorable outcome group and an mRS score of 2–6 forming the unfavorable outcome group [8,20].

Long-term outcome data were recorded as a combination of the neurological examination findings and the modified Rankin scale (mRS) and PSOM-SNE scores.

Informed consent was obtained from all of the families of the patients included in this study.

### 2.4. Ethical Approval

Approval for this study was obtained from the local ethics committee (12.06.2020-673).

### 2.5. Statistical Analysis

A statistical analysis was performed using Statistical Package for the Social Sciences (SPSS) 25th edition. The Kolmogorov–Smirnov test and Shapiro–Wilk tests were used to check for normal distributions. Mean, standard deviation, median, and minimum–maximum values were used in a descriptive analysis. Additionally, 2 × 2 tiles were compared with the Pearson Chi-square test, and Fisher’s exact test was used in selected situations. The Kruskal–Wallis test was used to compare two or more independent samples with a nonparametric distribution.

*p* value < 0.05 was considered statistically significant.

## 3. Results

A total of 32 patients (20 of whom were male, 62.5%) who met all the inclusion criteria and gave informed consent were included in this study. The mean age at first ischemic stroke was 77.39 ± 61.93 months. Age at first stroke was grouped as early (≤60 months) or late (>61 months), and 16 patients (50%) were included in the early group. The mean time that had passed from symptom onset to emergency room admission was 72.29 ± 158.2 h (median = 15.5). The mean follow-up duration of the study group was 85.44 ± 20.52 months (median = 84), and the mean age of the group at the time of the study (present age) was 162.62 ± 64.4 months. The demographic and clinical characteristics of the study group are presented in Table 1 and Table 2.

Focal motor weakness (42%) and facial asymmetry (19.4%) were the most common presenting signs.

All the patients had undergone diffusion-weighted imaging (DWI) MRI and cranial CT within the first two hours after hospital admission. Diagnostic cranial MRI and cranial/cervical MRI angiography were performed within the first 48 h after admission. MRI venography was performed on suspicion of venous thrombosis, and fat-suppressed T1 axial MRI images were obtained for patients with a history of trauma to detect cervical arterial hematomas. Conventional angiography (DSA) was performed in 13 patients (40.6%). The major indications for DSA were an undetermined etiology and findings of vasculopathy in the initial investigational studies.

The most commonly detected etiology was cardiac disease (37.3%), followed by moyamoya disease (21.8%). The etiology was not determined in 28.1% of patients despite all investigational studies.

Four of the six recurrent stroke cases had a cardiac etiology, and two of them had an undetermined etiology despite the detailed investigations (the genetic evaluation included a chromosome analysis, a CGH array, and WES (whole-exome sequencing)) (Table 2).

Long-term prognosis was the main target criterion in all statistical analyses in this study. The long-term clinical outcomes were grouped into four categories according to the neurological examination and family reports: Group 1, normal daily functions; Group 2, motor dysfunction; Group 3, cognitive dysfunction; and Group 4, motor and cognitive dysfunction. Group 1 was considered the best prognosis, and Group 4 was considered the worst prognosis. PSOM-SNE was scored between 0 and 10 points, and the results were also used for correlation with the clinical classifications. Motor deficit scored as 0–1 using the modified Rankin scale was included in the “normal daily function” group (Table 2). Motor deficit with a variable severity was detected in 13 patients (41%) at the last visit. Hemiparesis and/or hemiplegia was the neurological motor finding. All these patients were still receiving regular physiotherapy. Only 53.1% (*n* = 17) of the patients reported normal daily functions.

### 3.1. Stroke Age

The late stroke age group (>61 months) had a significantly higher rate of normal long-term functional outcomes (*p* = 0.023, Chi-square test) (Table 3).

When examining the PSOM-SNE scores of the 17 patients who reported having normal daily functions (Group 1), it was found that only 4 had zero points, and the remaining 13 had minimal abnormalities, specifically in language or cognitive and behavioral deficits, which had been missed by the neurological examination and parental questioning method.

The PSOM-SNE scores of the younger stroke age group (≤60 months) were significantly higher than those of the late age group (>61 months), and only two out of six patients with normal daily functions had zero points.

### 3.2. Hospital Admission Time Interval

The median time interval from the appearance of the first symptom to hospital admission was 24 (mean: 33.5 ± 14.2) hours in the younger stroke age group (≤60 months) and 9 (mean: 105.07 ± 208.4) hours in the late age group (>61 months). In both groups, patients who presented with nonspecific symptoms such as restlessness and an altered state of consciousness had longer hospital admission times.

### 3.3. Vascular Territory

We did not find a statistically significant difference between age at first stroke and vascular stroke territory (*p* = 0.176, Kruskal–Wallis test) (Table 4).

There was no statistical correlation between the presence of EEG abnormalities at admission and vascular stroke territory (*p* = 0.94, Chi-square test).

The mean time interval to hospital admission was compared according to vascular stroke territory, and a statistically significant difference was not found (*p* = 0.372, Kruskal–Wallis test).

Statistical differences could not be found in terms of presenting complaints at hospital admission according to vascular stroke territory (*p* = 0.401, Chi-square test).

Vascular territory also did not have any significant effect on long-term clinical outcomes (*p* = 0.550, Chi-square test).

Anterior circulation was the most common vascular territory (62.5%) in the early age group; however, although the late age group more commonly demonstrated a widespread stroke territory (56.25%), the difference between the groups was not statistically significant (*p* = 0.108, Chi-square test).

The initial treatment strategy was planned according to the etiology and risk factors rather than the affected vascular territory, and there was no significant difference according to the affected stroke territory between the treatment groups (*p* = 0.376, Chi-square).

### 3.4. Etiology

The stroke etiology did not have a statistically significant effect on the long-term outcomes (*p* = 0.088, Chi-square test).

### 3.5. Treatment Strategy

Patients receiving only anticoagulant treatment had statistically worse long-term outcomes in terms of neurologic sequalae than the patients receiving only antiplatelet treatment (*p* = 0.026) (Table 5). Aspirin was the only antiplatelet drug used in our study population. Subcutaneous enoxaparin was the preferred anticoagulant in all patients.

One patient who had a stroke in the hospital after cardiac surgery underwent an emergent thrombectomy in our study population. Afterward, his medical treatment only consisted of aspirin.

All patients stopped anticoagulant treatment after 6 months; however, patients with a cardiac etiology and moyamoya disease still received antiplatelet treatment regardless of their clinical condition.

## 4. Discussion

Childhood stroke is rare but time-critical, as the nonspecific initial symptoms and age-specific diagnostic challenges may delay early diagnosis. This could hinder the implementation of early brain-saving interventions, which have a critical impact on prognosis [1]. Since the publication of scientific evidence demonstrating that childhood stroke is not as harmless as believed for centuries, studies have focused on the development of new therapeutical interventions to improve its short- and long-term prognoses, as well as to decrease the lifetime burden of the related complications [21]. Since the discovery of the unique nature of pediatric stroke in terms of its etiology and treatment response, frequently updated expert consensus guidelines have played an important role in stroke management at well-equipped large centers. Initial stroke management in a well-organized center with an updated pediatric stroke protocol is likely to be advantageous in decreasing the mortality and morbidity of pediatric stroke.

The data presented in this study represent the long-term outcomes of pediatric patients whose initial assessment and follow-up were performed at an ideal tertiary clinic in line with the current consensus recommendations [22,23].

Cardiac disease and positive genetic tests for thrombophilia were found to be the most common risk factors for ischemic stroke in our population. Moyamoya disease and cardiac diseases were the two most common etiologies identified. This is consistent with the literature, as cardiac disease and arteriopathies have been defined as the two major risk factors for AIS in children [1,24]. One reason why cardiac diseases and moyamoya vasculopathy were found to be the most common etiologies in our study group could be because we only included patients who attended regular outpatient visits for more than 5 years. Having a chronic illness may motivate patients to attend regular neurology follow-ups despite being symptom-free.

Out of all the patients in our study, 18.7% (*n* = 6) had recurrent stroke attacks. Among these patients, two (33.3%) had moyamoya disease. This is consistent with the literature, where moyamoya disease was found to be responsible for pediatric AIS in 8% of cases and was related to higher recurrence rates [25]. Only one of our patients with an unknown etiology had a recurrent stroke under ongoing anticoagulation treatment. Otherwise, all patients receiving anticoagulation treatment had either stopped treatment or switched to antiplatelet treatment after 6 months. One consensus has advised antithrombotic therapy for two years after cryptogenic stroke [26]. Two of our patients were still receiving antiplatelet treatment and one patient was receiving anticoagulation treatment at the time of the stroke recurrence. Three of the patients with an unknown etiology stopped their medical treatment after becoming symptom-free, and they were not receiving any medical treatment at the time of recurrence.

Although several studies have revealed the short- and long-term outcomes of pediatric stroke, the retrospective nature of most of them and the variations in the study protocols remain the major reasons for the lack of clear conclusions [27,28].

Elbers et al. conducted one of the studies with the longest follow-up periods of childhood stroke, providing data on 26 pediatric ischemic stroke patients, with a mean follow-up time of 10 years and 10 months (with a range of 3–18 years), and 80% of the patients recovered completely or with only minimal sequelae. They pointed out that 25% of the symptoms were psychiatric, which was a relatively high ratio [29]. They also stressed that the PSOM-SNE scores in the first year of the event were the strongest predictor of long-term outcomes. In our study, PSOM-SNE was performed during the last visit for an outcome evaluation, and 76% of patients with normal daily functions and a normal neurological examination were found to have mild abnormalities in the PSOM-SNE scores. These abnormal scores enabled us to identify mild cognitive and language abnormalities and refer the patients for better treatment options [26]. Another study with a median follow-up time of 6.9 years after AIS in pediatric age reported favorable outcomes in 56% of cases [25].

Our study results revealed that a younger age at stroke insult was inversely correlated with good outcomes, and this finding is consistent with the literature [13,26]. Additionally, 42.9% of patients who had their first stroke under 60 months of age had normal daily functions in the fifth-year evaluation in our study. This finding is consistent with that of a study examining a high number of ischemic stroke patients, which revealed that 60% of the patients had abnormalities at different levels [26]. Stroke age has been found to be one of the most important prognostic factors for cognitive impairment [30]. In our study, we did not have the chance to perform sophisticated and detailed cognitive tests, but the PSOM-SNE scores were helpful in detecting some mild cognitive and language dysfunctions that did not interfere with daily life.

The median time interval from the first symptom to hospital admission was 15.5 (1–720) hours in our study group, which is shorter than that in a study conducted by Rafay et al., who reported a median time interval of 22.7 h in pediatric stroke patients [31]. However, some studies have also reported this time as 1.3 to 10.5 h, which is more acceptable for the time-critical period of acute intervention [27]. One reason for this striking delay in arrival at our clinic could be because our hospital is not centrally located; additionally, the use of public transport is not feasible after midnight, thus presenting another factor as to why our clinic may not have been the first place of arrival after acute pediatric stroke. This indicates the importance of providing centrally located hospitals with good stroke intervention teams, as it would probably increase the survival rate of acute pediatric ischemic stroke. However, although some studies presented no delay in hospital admission time (90% within 24 h), there were still contradictions in first-line treatment plans, and the long-term survival rates were still not as optimistic as expected [31].

The younger age subgroup (≤60 months) in our study had a longer hospital admission time (24 h vs. 9 h). This could be attributed to the higher ratio of nonspecific symptoms at admission in this age group, which is one of the main reasons for the diagnostic delay of acute ischemic stroke in pediatric patients [30]. Stroke territory was not found to affect the hospital admission time in our study; this is in contrast to other studies in the literature, which found that posterior circulation was related to a longer hospital admission time [30]. One reason for this difference could be because of the relatively low number of patients with only posterior circulation stroke (6.3%) in our study. Antiplatelet therapy alone was the most preferred treatment modality, and this is consistent with the literature considering that the most common etiologies were cardiac diseases and vasculopathies in our population [1]. However, the best treatment strategy for acute arterial ischemic stroke in the pediatric population remains unknown and depends on the knowledge and previous experience of the clinic [1].

### Study Limitations

The aim of this study was to examine the long-term outcome data of patients who had been followed by the same clinic, starting from admission and continuing throughout the entire treatment process. Patients with worse symptoms at presentation who needed treatment in an intensive care unit and who were referred to our clinic after an initial medical intervention in another clinic were excluded from this study to rule out potential confounding factors that may have affected long-term prognosis. Patients who did not regularly attend follow-up visits or continued their follow-ups at different clinics were also excluded. This limited the number of patients included in this study.

PSOM-SNE (over 2 years) was performed in all patients, as it allowed for the objective scoring of signs and symptoms in five main areas. However, some of the items on this scale require patient cooperation, and this may have caused a bias in the results of patients who did not pay full attention.

The PedNIHSS scores of some patients at admission and follow-up were missing, and the parameters were not included in the study, so short-term outcome data were not given. Medical data recorded in the emergency room did not provide enough information on the exact time interval between the first treatment and hospital admission time. Additionally, the patients’ caregivers had difficulty in recalling this information retrospectively, as a long time had passed since the event. These data would have increased the value of the present study. This also indicates the importance of detailed and precise data recording in addition to rapid interventions in accordance with the current guidelines by a multidisciplinary experienced pediatric emergency stroke team.

## 5. Conclusions

Although this study examined a limited number of patients, it is unique in terms of its focus on the long-term outcomes of AIS pediatric patients with a favorable initial clinical presentation. The study results show that, even when followed by a well-experienced clinic, early stroke age has a high likelihood of leading to long-term neurological sequalae. Easily applicable practical scales that have been developed and validated for measuring stroke outcomes in children are useful for the early detection of occult neurological abnormalities that can be missed by sole neurological examinations, thus allowing for appropriate early interventions. PSOM-SNE is an easily applicable test that can be used in routine stroke follow-up in tertiary stroke centers with a relatively large number of pediatric stroke patients.

## Figures and Tables

**Table 1:** Baseline characteristics of the study group.

		Mean ± s.s	Median (Min–Max)
Age at first stroke (months)		77.39 ± 61.93	66 (0.5–180)
Time interval to hospital admission (h)		72.29 ± 158.2	15.5 (1–720)
Follow-up interval (months)		85.44 ± 20.52	84 (60–121)
Present age (months)		162.62 ± 64.4	142.5 (62–300)
		N (%)	
Age at first stroke (group)	≤60 months	16 (50)	
	>61 months	16 (50)	
* Presence of EEG abnormality		14 (43.7)	
Regular physiotherapy treatment		24 (75)	
Recurrence of stroke		6 (18.7)	

EEG: electroencephalogram * focal epileptiform discharges, slow background activity, hemispheric asymmetry in background activity.

**Table 2:** Clinical characteristics of the study group.

		N (%)
Presenting complaint (symptom/sign)	Restlessness	3 (4.8)
	Hemiparesis and/or hemiplegia	26 (42)
	Aphasia	3 (4.8)
	Seizure	7 (11.3)
	Altered state of consciousness	3 (4.8)
	Facial asymmetry	12 (19.4)
	Ataxia	5 (8.1)
	Memory loss	2 (3.2)
	Vertigo	1 (1.6)
	Total	62 (100)
Etiology	Unknown	9 (28.1)
	Cardiac disease	5 (15.6)
	Moyamoya disease	7 (21.9)
	Thrombophilia	5 (15.6)
	Infection (VZV)	1 (3.1)
	Trauma	1 (3.1)
	CNS aneurysm	1 (3.1)
	Genetic dysmorphism	2 (6.3)
	Total	32 (100)
Risk factor	Genetic syndrome (Down syndrome)	1 (2.2)
	Cardiac disease (total)	12 (26)
	Surgery for secundum ASD	1
	Surgery for tetralogy of fallout	3
	Fontan surgery for hypoplastic left heart	1
	Dilated cardiomegaly secondary to parvovirus infection	1
	Surgery for pulmonary stenosis	1
	Surgery for coarctation of aorta	1
	Mitral valve disease secondary to ARF	1
	Patent foramen ovale	2
	Bicuspid aorta	1
	Pathological tests for hypercoagulability (total)	32 (69.6)
	PAI gene homozygous mutation	3
	Low protein C	3
	High Lipoprotein A	2
	Low Factor 8	1
	MTHFR C677T heterozygous mutation	9
	MTHFR A1298C heterozygous mutation	8
	Factor V Leiden heterozygous mutation	1
	High homocysteine level	4
	High anti-thrombin 3 level	1
	Family history of stroke	1 (2.2)
	Total	46 (100)
Radiological localization territory of ischemic infarct	Anterior	17 (53.1)
	Posterior	2 (6.3)
	Widespread	13 (40.6)
	Total	32 (100)
Vascular localization of ischemic stroke	MCA	16 (50)
	ICA	1 (3.1)
	PCA	1 (3.1)
	Vertebral artery	1 (3.1)
	Multiple vessels	13 (40.7)
	Total	32 (100)
Treatment modality	Antiplatelets (aspirin)	9 (28.2)
	Anticoagulants	5 (15.6)
	Both antiplatelets (aspirin) and anticoagulants	8 (25)
	Initial anticoagulants, followed by antiplatelets	8 (25)
	Acute thrombectomy, followed by antiplatelets (aspirin)	1 (3.1)
	No medical treatment	1 (3.1)
	Total	32 (100)
Long-term clinical outcome	Normal daily functions	17 (53.1)
	Motor dysfunction	6 (18.8)
	Cognitive dysfunction	2 (6.2)
	Motor and cognitive dysfunction	7 (21.9)

VZV: varicella zoster virus; MCA: middle cerebral artery; ICA: internal carotid artery; PCA: posterior cerebral artery; CNS: central nervous system; ARF: acute rheumatic fever; ASD: atrial septal defect.

**Table 3:** Relationship between long-term outcomes and stroke age.

		Stroke Age Group				*p* **
		≤60 Months		>61 Months		
		*n*	%	*n*	%	
Long-term clinical outcome	Normal daily functions	6	(37.5)	11	(61.1)	0.023
	Motor dysfunction	2	(12.5)	4	(22.2)	
	Cognitive dysfunction	1	(6.3)	1	(5.6)	
	Motor and cognitive dysfunction	7	(43.7)	2	(11.1)	
PSOM-SNE scores (mean ± SD)		16	2.25 ± 2.48	16	1.18 ± 0.65	0.018

** Chi-square test.

**Table 4:** Stroke age and vascular territory.

		Stroke Age (Months)		*p* *
		Mean ± SD	Median (Min–Max)	
Stroke vascular territory	Anterior	66.97 ± 66.41	48 (0.5–180)	0.176
	Posterior	25.5 ± 23.33	25.5 (9–42)	
	Widespread	99 ± 53.61	94 (2–180)	

* Kruskal–Wallis test.

**Table 5:** Relationship between initial medical treatment and long-term outcomes.

Treatment *		Neurological Sequelae		*p* **
		No	Yes	
Only antiplatelet	*n*	8	1	0.026
	%	88.9%	11.1%	
Only anticoagulant	*n*	1	4	
	%	20.0%	80.0%	
Both antiplatelet and anticoagulant	*n*	8	8	
	%	50.0%	50.0%	

* Eight patients who were first started on an anticoagulant and continued with only an antiplatelet were included in the “both antiplatelet and anticoagulant” group. Two patients who did not receive any treatment were not included in the statistical analysis. ** Chi-square test.

## Data Availability

All the data supporting the findings of the present study can be provided by the first author upon reasonable request due to privacy.
